# 气相色谱-质谱法测定土壤中14种苯胺类和联苯胺类化合物

**DOI:** 10.3724/SP.J.1123.2023.01002

**Published:** 2023-12-08

**Authors:** Lijuan WU, Lili YANG, Enyu HU, Meifei WANG, Chao YANG, Mingming YIN

**Affiliations:** 1.江苏省南京环境监测中心,江苏南京210041; 1. Nanjing Environmental Monitoring Centre, Nanjing 210041, China; 2.江苏省环境监测中心,江苏南京210041; 2. Jiangsu Environmental Monitoring, Nanjing 210041, China

**Keywords:** 气相色谱-质谱, 苯胺类化合物, 联苯胺类化合物, 土壤, gas chromatography-mass spectrometry, aniline compounds, benzidine compounds, soil

## Abstract

针对土壤基质复杂及部分苯胺类化合物提取效率低的特点,基于气相色谱-质谱建立了土壤中14种苯胺类和联苯胺类化合物的分析方法。在采集后的土壤样品中加入5%亚硫酸钠水溶液进行密封,以防止目标物氧化变质,并于4 ℃以下冷藏保存,保存期限可达7天。实验比较了加速溶剂提取和振荡分散提取两种方式的提取效率,最终采用提取时加入碱性水溶液和乙酸乙酯-二氯甲烷(1∶4, v/v)的方法,在土壤-水相-有机相共存的条件下进行振荡分散萃取。萃取完成后经离心弃去土壤固相,将水相和有机相转移至分液漏斗中,并分离出有机相,再经弗罗里硅土固相净化柱净化、浓缩后,采用气相色谱-质谱联用仪测定,并以苯胺-d_5_和苊-d_10_为内标进行定量分析。实验考察了抗氧化剂和萃取溶剂种类、有机相与水相比例、盐析等对目标物萃取效率的影响,并在优化的实验条件下考察了方法的回收率及精密度。结果表明,14种目标化合物在0.5~100 mg/L范围内有良好的线性关系,相关系数(*R*)>0.999,方法检出限和定量限分别为0.02~0.07 mg/kg和0.08~0.28 mg/kg,在实际样品中的加标回收率为62.9%~101.0%, 6次精密度试验的相对标准偏差为3.8%~10.3%。利用所建立方法对江苏某工业企业疑似苯胺污染地块的土壤进行了检测,共检出两种苯胺类化合物。本方法简便、稳定、回收率高,特别是能够有效抑制苯胺类和联苯胺类化合物的氧化,可用于土壤中苯胺类和联苯胺类化合物的检测。

## Abstract (en)

The complex matrix of soil samples and low extraction efficiency of aniline compounds limit many methods developed for detecting aniline and benzidine compounds in soil. In this study, a rapid and sensitive method based on gas chromatography-mass spectrometry was developed for the simultaneous determination of 14 aniline and benzidine compounds in soil. The collected soil samples were sealed with 5% sodium sulfite solution and refrigerated to inhibit the oxidation of the target compounds for up to 7 d. The extraction efficiencies of accelerated solvent extraction and oscillating dispersion extraction were compared, and the recovery of accelerated solvent extraction was found to be unsuitable. Hence, three-phase oscillating dispersion extraction was adopted. A certain amount of alkaline aqueous solution was added to the test system during extraction to improve the extraction efficiency because aniline and benzidine compounds are weakly alkaline substances. When the pH of the extracted water phase was greater than 12, relatively good recoveries were obtained. Next, a mixed solvent of ethyl acetate-methylene chloride (1∶4, v/v) was added to extract the target compounds via oscillation for 20 min. The solid phase was discarded via centrifugation, and the aqueous and organic phases were transferred to a liquid separation funnel for further separation. Finally, the organic phase was retained. This pretreatment process prevents the co-extraction of acidic compounds or other impurities, thereby enhancing the purification ability of the method. Solid phase extraction (SPE) is generally recommended for soil extraction and purification. A preliminary test showed that compared with other columns, the Florisil SPE column could better retain the target substances and exhibited higher elution efficiency. After purification, the organic phase was concentrated to 1 mL using a nitrogen blower. The analytes were analyzed by gas chromatography-mass spectrometry using a capillary column (DB-35MS, 30 m×0.25 mm×0.25 μm). The temperature program was optimized to separate the target compounds at the baseline. Specifically, the initial oven temperature was set to 60 ℃, held for 2 min, increased to 130 ℃ at a rate of 5 ℃/min, increased to 300 ℃ at a rate of 30 ℃/min, and held for 4 min. The injector and ion source temperatures were 250 and 300 ℃, respectively. Aniline-d_5_ and acenaphthene-d_10_ were used as the internal standards for quantification. The effects of antioxidant addition, extraction solvent type, salting out, and other factors on extraction efficiency were investigated. The results showed that the method performed well under the optimized experimental conditions when actual soils were used as real sample matrices. The accuracy and precision of the proposed method were verified. A total of 14 aniline and benzidine compounds demonstrated good linearities in the range of 0.5-100 mg/L. The method detection limits (MDLs) ranged from 0.02 to 0.07 mg/kg, and the limits of quantification (LOQs) ranged from 0.08 to 0.28 mg/kg. The target compounds were spiked at contents of 1 and 10 mg/kg. The spiked recoveries of the 14 targets in actual soils were 62.9%-101%, and the relative standard deviations (RSDs) of six precision tests were 3.8%-10.3%. The proposed method effectively inhibited the oxidation of aniline and benzidine compounds during extraction, and the target compounds exhibited high recoveries and good stabilities in the presence of three phases. Moreover, the operating procedure was simple and easy to implement. The proposed method was applied to the soil collected from an industrial enterprise in Jiangsu province that was suspected to be contaminated with aniline, and two aniline compounds were detected. The developed method requires a small sample size, and the preservation step is simple and effective. In addition, it can be applied to various types of actual soils. The method meets the requirements of current soil pollution risk control standards for aniline and benzidine compounds in soils.

苯胺类和联苯胺类化合物均属于芳香胺类化合物,被广泛应用于医药、农药、染料、树脂、橡胶、塑料、清漆等药物和工业产品的生产过程,同时也是杀虫剂、高分子材料、炸药等重要化工产品的合成原料。近年来在大气、地下水、河流、土壤和底泥中已发现苯胺类和联苯胺类化合物的残留,这类污染物含量低、分布广、毒性大,对环境和生物构成极大危害。部分苯胺类和联苯胺类化合物被报道具有“三致”(致癌、致畸、致突变)效应,如苯胺和联苯胺已被世界卫生组织国际癌症研究机构列为致癌物^[1]^。我国针对苯胺类及联苯胺类化合物的控制主要集中于水质和食品等,目前仅在《土壤环境质量 建设用地土壤污染风险管控标准(试行)》(GB 36600-2018)^[2]^中规定了土壤中苯胺和3,3'-二氯联苯胺的筛选值与管制值。

苯胺类和联苯胺类化合物分子中的氨基含有孤对电子,其反应活性较高^[3]^,在复杂的土壤基质中检测难度较大。现行的《土壤和沉积物 13种苯胺类和2种联苯胺类化合物的测定 液相色谱-三重四极杆质谱法》(HJ 1210-2021)采用内标校正法进行结果计算,该方法所选择的内标指示物需要与目标化合物相匹配,并具有相近的加标回收率,否则会直接影响定量结果^[4]^。文献方法大多采用经典的索氏提取、加速溶剂提取(ASE)等液固提取方法^[5⇓⇓⇓⇓-10]^,检测手段有气相色谱法、气相色谱-质谱法、液相色谱-质谱法等^[11⇓⇓⇓⇓-16]^。苯胺类和联苯胺类化合物容易被氧化,采样后的保存问题对其后期的提取与测定有着明显的影响,但目前在土壤介质中多种苯胺类和联苯胺类化合物的分析中未见提及。

本文通过直接将土壤样品保存于还原性盐水溶液中,全部用于后期提取,再将目标化合物从固相转移至水相再转移至有机相中,一并解决了样品的保存、抗氧化、土壤含水率影响等难题。该方法样品保存效果好,操作简单,精密度和回收率能够满足实际样品的测定要求。

## 1 实验部分

### 1.1 仪器、试剂与材料

7890B-5977A气相色谱-质谱仪(美国安捷伦科技公司),配备电子轰击(EI)电离源;频率可调的ZP-500水平振荡装置(上海华邻实业有限公司); TGL16M台式离心机(长沙湘智离心机仪器有限公司); AutoVap S8 Plus氮吹浓缩仪(美国ATR公司); XW-80A涡旋振荡器(海门市其林贝尔仪器制造有限公司)。

乙酸乙酯、二氯甲烷、甲醇(色谱纯,德国Merck公司),弗罗里硅土固相净化柱(1 g/6 mL,美国Supelco公司),实验用水由Milli-Q超纯水系统(美国Millipore公司)制备,其余试剂均为市售分析纯。

苯胺类和联苯胺类化合物单标溶液,包括苯胺、4-甲基苯胺、2-甲基苯胺、3-甲基苯胺、2,4-二甲基苯胺、2,6-二甲基苯胺、2-甲氧基苯胺、3-氯苯胺、4-氯苯胺、3-硝基苯胺、2-萘胺、4-硝基苯胺、联苯胺、3,3'-二氯联苯胺共14种组分,5000 mg/L,购自北京曼哈格生物科技有限公司;用甲醇将以上单标溶液配制成质量浓度为500 mg/L的混合标准工作溶液。内标苯胺-d_5_和苊-d_10_(2000 mg/L),购自美国Accustandard公司;用甲醇将两种内标配制成质量浓度为500 mg/L的混合内标标准溶液。替代物4-氯苯胺-d_2_,购自加拿大CDN Isotopes公司;在精确称量后用甲醇溶解,并配制成质量浓度为500 mg/L的替代物标准溶液。所有标准溶液在4 ℃下密闭避光保存。

### 1.2 工作溶液的配制

移取适量的14种苯胺类和联苯胺类混合标准工作溶液及替代物标准溶液,用乙酸乙酯-二氯甲烷(1∶4, v/v)配制成质量浓度为0.5、1.0、5.0、10、50、100 mg/L的系列混合标准溶液。向系列混合标准溶液中分别加入适量混合内标标准溶液,配制成内标化合物质量浓度为10 mg/L的系列工作溶液。

### 1.3 样品采集和前处理

#### 1.3.1 样品采集

在40 mL棕色具塞玻璃样品瓶中预先加入20 mL 5%亚硫酸钠水溶液,采集约10 g土壤样品装入瓶中,盖紧盖子,上下振荡使溶液覆盖所有样品,并于4 ℃以下密闭避光保存。

#### 1.3.2 样品提取

将样品瓶在涡旋振荡器上进行涡旋分散,直至样品在5%亚硫酸钠水溶液中呈泥浆状,之后将样品全部转移至锥形瓶中;用10 mL 3 mol/L的氢氧化钠溶液分两次荡洗样品瓶,将荡洗溶液合并至锥形瓶中;在锥形瓶中加入10 μL替代物标准溶液和2 g氯化钠,再加入30 mL乙酸乙酯-二氯甲烷(1∶4, v/v),置于水平振荡装置中,振荡频率保持在250 r/min以上,以保证三相能够完全均匀分散;振荡20 min后再全部转移至100 mL离心管中,以3000 r/min离心10 min,将液相部分全部转移至分液漏斗中,并分离出有机相;向分液漏斗中加入30 mL乙酸乙酯-二氯甲烷(1∶4, v/v)再进行一次提取,合并有机相,并用无水硫酸钠脱水后,将提取液浓缩至约1 mL,加入5 mL正己烷,待净化。

#### 1.3.3 样品净化

将弗罗里硅土固相净化柱分别用5 mL二氯甲烷和5 mL正己烷冲洗、平衡,待净化柱充满正己烷后关闭流速控制阀,浸润5 min,缓慢打开控制阀,弃去流出液。待正己烷流至近干时,将待净化的提取液转移至净化柱内,弃去滤液,用10 mL乙酸乙酯-二氯甲烷(1∶4, v/v)洗脱,接收洗脱液并浓缩至约1 mL,加入混合内标标准溶液,使内标质量浓度为10 mg/L,再用乙酸乙酯-二氯甲烷(1∶4, v/v)定容至1 mL,待测定。

### 1.4 仪器条件

#### 1.4.1 色谱条件

Agilent DB-35MS毛细管色谱柱(30 m×0.25 mm×0.25 μm);柱温60 ℃,保持2 min,以5 ℃/min升温至130 ℃,然后以30 ℃/min升温至300 ℃,保持4 min;进样口温度250 ℃;载气为高纯氦气(99.999%),柱内流量采用恒流控制,流速1.0 mL/min;分流进样,分流比5∶1,进样量1 μL。

#### 1.4.2 质谱条件

离子源:EI源;离子源温度:300 ℃;离子化能量:70 eV;接口温度:300 ℃;四极杆温度:150 ℃;质谱扫描范围*m/z* 45~550;溶剂延迟时间:5 min;数据采集模式:全扫描(Scan)。14种目标化合物的质谱参数见表1。

**表1:** 14种目标化合物的质谱参数

Compound	Quantitative ion (*m/z*)	Auxiliary qualitative ions (*m/z*)
Aniline (苯胺)	93	94, 66
*p*-Toluidine (4-甲基苯胺)	106	107, 77
*o*-Toluidine (2-甲基苯胺)	106	107, 77
*m*-Toluidine (3-甲基苯胺)	106	107, 79
2,4-Xylidine (2,4-二甲基苯胺)	121	106, 120
2,6-Xylidine (2,6-二甲基苯胺)	121	106, 77
*o*-Anisidine (2-甲氧基苯胺)	108	123, 80
*m*-Chloroaniline (3-氯苯胺)	65	127, 129
*p*-Chloroaniline (4-氯苯胺)	127	129, 65
*m*-Nitroaniline (3-硝基苯胺)	92	138, 65
2-Naphthalenamine (2-萘胺)	143	115, 116
*p*-Nitroaniline (4-硝基苯胺)	65	138, 105
Benzidine (联苯胺)	184	156, 92
3,3'-Dichlorobenzidine	252	254, 154
(3,3'-二氯联苯胺)		
Aniline-d_5_(苯胺-d_5_)	98	71
Acenaphthene-d_10_(苊-d_10_)	162	164, 160
*p*-Chloroaniline-d_2_(4-氯苯胺-d_2_)	129	67, 131

## 2 结果与讨论

### 2.1 色谱条件的优化

由于苯胺类和联苯胺类化合物具有一定的极性,两对同分异构体(2-甲基苯胺和4-甲基苯胺、2,4-二甲基苯胺和2,6-二甲基苯胺)在弱极性的毛细管柱上无法分开,且其余组分的峰形存在拖尾现象。选择中等极性色谱柱,在优化的柱温条件下,14种目标化合物可以得到良好的分离,且峰形更加对称和尖锐。14种苯胺类和联苯胺类化合物的总离子流色谱图见图1。

**图1:**
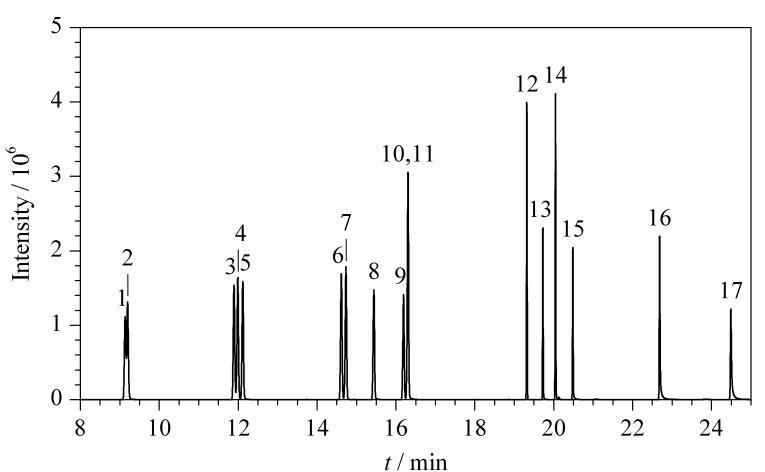
14种苯胺类和联苯胺类化合物、替代物及内标的 总离子流色谱图

### 2.2 样品前处理条件的优化

#### 2.2.1 样品提取方法的选择

土壤中有机污染物常用的提取方法有索氏提取、微波提取、加速溶剂提取、超声波提取等,这些方法都是利用有机溶剂对目标物的溶解进行提取。为了消除土壤基质中水分的影响,常用风干法或化学试剂干燥法对土壤样品进行脱水。由于苯胺类和联苯胺类化合物的分子结构中存在氨基,增大了目标物的极性和不稳定性,常用的提取方法在干燥和提取操作过程中受水分含量、氧化降解的影响较大,提取效率不高。

近年来,加速溶剂提取是土壤中有机污染物提取应用较多的方式,其具有快速、高效的优势,在很多研究和标准方法中都有着广泛的应用^[17,18]^。实验比较了各目标化合物采用不同提取溶剂经ASE提取后的回收率,结果发现,使用二氯甲烷、甲醇、四氢呋喃、乙酸乙酯及丙酮的提取效果均不理想。分析其原因,一方面,土壤基质对苯胺类和联苯胺类化合物有着较强的吸附能力,仅使用常用的有机溶剂进行提取时效果均不理想;另一方面,苯胺类化合物在空气中容易被氧化,在提取过程中会发生降解,因而导致回收率偏低。为了解决以上两个问题,在土壤样品中同时加入碱性水相和提取有机相,通过激烈振荡使得三相充分分散混匀,将土壤中所含苯胺类和联苯胺类化合物从水相转移至有机相中,14种目标化合物均获得了较好的提取效果,不同提取方式下14种目标化合物的回收率结果见表2。此外,实验比较了硫代硫酸钠、亚硫酸钠和抗坏血酸的抗氧化效果,加入硫代硫酸钠和抗坏血酸后,目标化合物的回收率分别为66.2%~86.5%和69.6%~92.0%,加入亚硫酸钠后目标化合物的回收率为80.5%~90.5%,可见在提取的同时加入抗氧化剂能够有效防止目标化合物的氧化降解。

**表2:** 不同提取方式下14种目标化合物的回收率

Compound	ASE recoveries/%							Three-phase dispersion recovery/%
	CH_2_Cl_2_	CH_3_OH	C_4_H_8_O	CH_2_Cl_2_ -CH_3_COCH_3_ (1∶1, v/v)	CH_2_Cl_2_-C_4_H_8_O (1∶1, v/v)	CH_2_Cl_2_-CH_3_COOC_2_H_5_ (1∶1, v/v)	CH_3_COCH_3_	
Aniline	43.1	17.6	1.0	32.8	5.7	7.9	56.9	80.0
*p*-Toluidine	37.5	11.3	2.1	31.6	8.9	10.4	46.2	80.6
*o*-Toluidine	38.1	10.2	1.9	32.0	9.1	9.8	48.1	80.7
*m*-Toluidine	37.4	12.9	4.0	35.4	3.3	4.6	53.0	80.9
2,4-Xylidine	37.5	6.8	11.6	37.3	22.0	25.4	36.8	81.1
2,6-Xylidine	35.2	5.8	10.2	35.6	20.1	23.5	35.6	80.0
*o*-Anisidine	39.3	1.8	2.1	33.1	5.0	9.6	26.5	80.7
*m*-Chloroaniline	59.0	29.0	2.1	53.8	7.7	8.4	58.6	80.5
*p*-Chloroaniline	55.4	28.3	1.8	35.8	6.4	7.8	52.1	80.5
*m*-Nitroaniline	95.4	83.8	0.9	107.0	21.3	20.2	50.8	82.4
2-Naphthalenamine	94.2	22.1	1.0	71.9	2.1	1.9	24.8	83.6
*p*-Nitroaniline	94.1	103.3	2.5	64.9	49.8	46.4	84.1	84.6
Benzidine	2.2	14.7	0.5	50.2	0.5	0.5	12.6	83.1
3,3'-Dichlorobenzidine	37.6	14.1	0.5	61.1	5.3	6.4	32.4	85.5

ASE: accelerated solvent extraction; CH_2_Cl_2_: dichloromethane; CH_3_OH: methanol; C_4_H_8_O: tetrahydrofuran; CH_3_COCH_3_: acetone; CH_3_COOC_2_H_5_: ethyl acetate.

#### 2.2.2 样品采集和保存方法的优化

苯胺类化合物具有易氧化的特性,因此样品在采集后需要合适的保存方式来抑制目标化合物的氧化。一般密闭冷藏保存的氧化抑制效果较差,HJ 1210-2021中提到,在4 ℃以下冷藏保存,大部分化合物可延长保存期限至24 h,冷冻保存化合物可延长保存期限至4~28 d。虽然冷冻保存可延长保存期限,但对于现场采样后需要立即冷冻的要求,操作上非常不便,所以保存方法优化实验均在4 ℃以下冷藏进行。实验首先考察了样品直接冷藏、加入固体亚硫酸钠搅拌后冷藏及利用纯水隔绝空气后冷藏的保存效果。结果发现,利用纯水隔绝空气的方式进行冷藏保存时,样品的抗氧化效果较好,明显优于直接冷藏或加入固体亚硫酸钠搅拌后冷藏。但这3种条件在冷藏保存了24 h及96 h内,仍然存在部分组分回收率过低的情况。在提取过程的优化中,加入亚硫酸钠的抗氧化效果最佳,因此在隔绝空气的基础上,考察了20%和5%亚硫酸钠水溶液作为保存剂时对14种目标化合物回收率的影响。结果表明,二者抗氧化效果相当,但由于20%亚硫酸钠水溶液为近饱和溶液,其溶解度受温度影响较大且需超声溶解,最终采用5%亚硫酸钠水溶液直接在采样现场加入的方式,保存期限可达7天,可以一并解决保存和后续的提取问题。不同保存方式下14种目标化合物和替代物的回收率见表3。在此实验基础上,以40 mL棕色具塞玻璃样品瓶作为容器,加入20 mL 5%亚硫酸钠水溶液,现场采集约10 g样品加入其中,在低于4 ℃的条件下进行运输和冷藏保存,在样品提取时全部转移至提取容器中。

**表3:** 不同保存方式下14种目标化合物和替代物的回收率

Compound	Recoveries/%											
	Refrigerate		Refrigerate with 2 g Na_2_SO_3_			Pure water			5% sodium sulfite aqueous solution			
	24 h		24 h			24 h	96 h		24 h		72 h	7 d
Aniline	74.2		71.2			84.8	87.1		91.9		88.7	86.5
*p*-Toluidine	80.6		94.1			85.9	88.0		92.9		83.8	87.1
*o*-Toluidine	57.7		72.2			86.7	89.9		92.6		84.9	85.2
*m*-Toluidine	64.2		78.2			87.0	90.3		93.1		84.6	85.5
2,4-Xylidine	50.2		97.0			86.5	92.8		94.3		85.0	87.6
2,6-Xylidine	70.3		107.0			86.7	92.9		93.6		85.5	84.8
*o*-Anisidine	33.0		90.3			86.2	89.1		95.3		86.2	86.4
*m*-Chloroaniline	93.5		107.0			86.8	95.4		97.4		87.2	85.0
*p*-Chloroaniline	88.8		102.0			86.0	95.0		97.2		86.4	85.6
*m*-Nitroaniline	87.6		45.1			86.3	102.0		83.2		75.1	77.0
2-Naphthalenamine	44.3		33.9			84.6	85.3		86.4		81.8	86.2
*p*-Nitroaniline	101.0		110.0			87.5	102.0		99.6		89.9	86.4
Benzidine	61.3		93.8			102.0	34.3		105.0		89.6	86.5
3,3'-Dichlorobenzidine	93.8		87.2			99.1	105.0		99.2		94.3	90.1
*p*-Chloroaniline-d_2_	83.2		98.6			88.1	105.0		89.4		88.4	90.4

#### 2.2.3 提取溶剂及相比的选择

苯胺类和联苯胺类化合物多为弱碱性物质,在碱性条件下萃取效率较高,因此提取时需保证水相部分pH>12。土壤基质中含有各种盐类,具有一定的缓冲能力,通过试验加入碱性溶液的体积和浓度,最后确定加入10 mL 3 mol/L的NaOH溶液,有利于将苯胺类化合物分配转移至有机相中。实验还考察了二氯甲烷和不同体积比的乙酸乙酯-二氯甲烷(2∶1、1∶4, v/v)对14种目标化合物的提取效果。结果表明,乙酸乙酯-二氯甲烷(1∶4, v/v)的提取效果最佳,不仅提取效率高,且易于分离和浓缩。由于采样环节包含了20 mL的水相部分,为了保证有机相的萃取效果和分离的方便性,应尽量控制两相的比例和总体积。因此,最终确定加入30 mL乙酸乙酯-二氯甲烷(1∶4, v/v),其与水相的体积比约为1∶1,用100 mL离心管分离。由于不同目标化合物在相与相之间的分配系数存在差异,实验发现个别极性较强的化合物经一次提取后的回收率较低,为了简化操作步骤,仅增加对水相的二次萃取,可使各目标化合物的回收率达到70%以上。

#### 2.2.4 盐析作用的影响

由于土壤含有一定的有机质和缓冲盐,在碱性条件下分散提取时可能产生乳化现象,加入氯化钠可增加水相溶液的离子强度,通过盐析作用降低目标物的溶解度,从而使14种目标化合物的回收率得到明显提高。实验结果表明,加入2 g以上的氯化钠能达到较好的盐析效果,离心分离弃去土壤固相后,剩余的水相和有机相分层更加明显,14种目标化合物和替代物的回收率结果见表4。

**表4:** 盐析对14种目标化合物和替代物回收率的影响

Compound	Recoveries/%	
	Without NaCl	With 2 g NaCl
Aniline	71.2	82.2
*p*-Toluidine	70.8	81.9
*o*-Toluidine	73.5	81.7
*m*-Toluidine	76.5	82.1
2,4-Xylidine	76.9	81.8
2,6-Xylidine	76.2	80.4
*o*-Anisidine	73.6	81.6
*m*-Chloroaniline	72.1	80.8
*p*-Chloroaniline	71.2	81.0
*m*-Nitroaniline	75.3	95.8
2-Naphthalenamine	78.9	99.1
*p*-Nitroaniline	76.3	99.7
Benzidine	72.0	106.0
3,3'-Dichlorobenzidine	77.4	111.0
*p*-Chloroaniline-d_2_	78.2	88.9

#### 2.2.5 样品净化方式的选择

本方法采用在碱性条件下进行样品萃取的方式,以实现目标化合物的选择性萃取,并起到一定的净化作用。萃取所得到的浓缩液颜色明显比ASE萃取浓缩液浅,通过比较全扫描质谱图发现,杂质出峰数量也明显减少,基线响应低。虽然土壤基质复杂,采用质谱检测器选择定量离子进行积分仍然可以较好地实现目标化合物的定性和定量,但对于有机质含量特别高的土壤,连续地对其进行进样分析,萃取液中的高沸点杂质可能会影响色谱柱性能,特别是影响极性较大的目标物的峰形,进而干扰测定。针对上述问题,选择固相净化小柱对萃取液进行净化^[19,20]^,通过筛选C18、弗罗里硅土、HLB等常用净化柱发现,C18与HLB柱对部分目标化合物的保留效果较差,最终确定先在萃取浓缩液中加入正己烷调节极性,之后利用弗罗里硅土净化柱对萃取液进行净化。实验比较了乙酸乙酯-二氯甲烷(1∶4, v/v)、二氯甲烷、甲醇等洗脱溶剂对14种目标化合物回收率的影响,最终选择乙酸乙酯-二氯甲烷(1∶4, v/v)作为洗脱溶剂,以避免进样溶剂歧视效应,净化后14种目标化合物的回收率为88.3%~99.5%。

### 2.3 方法学验证

#### 2.3.1 校准曲线、方法检出限和定量限

采用优化的方法分析系列质量浓度的工作溶液,绘制校准曲线。按照目标化合物总离子流色谱图的出峰顺序,苊-d_10_之前出峰的目标物以苯胺-d_5_为内标,苊-d_10_之后出峰的目标物以苊-d_10_为内标。

参照《环境监测分析方法标准制订技术导则》(HJ 168-2020)^[21]^确定方法检出限(MDL)和定量限(LOQ),在10 g空白石英砂中加入苯胺类和联苯胺类混合标准工作溶液,使目标物的含量为0.1~0.2 mg/kg。按照样品分析步骤,进行7次空白加标平行试验,并对结果进行统计计算。结果显示,在0.5~100 mg/L内各目标化合物的线性相关系数(*R*)均>0.999, MDL为0.02~0.07 mg/kg, LOQ为0.08~0.28 mg/kg,其中LOQ约为MDL的4倍,相关结果见表5。

**表5:** 14种目标化合物及替代物的校准曲线、相关系数、 方法检出限和定量限

Compound	Calibration curve	*R*	MDL/ (mg/kg)	LOQ/ (mg/kg)
Aniline	*y*=1.123*x*+0.005	0.9999	0.03	0.12
*p*-Toluidine	*y*=1.344*x*-0.167	0.9997	0.02	0.08
*o*-Toluidine	*y*=1.090*x*+0.150	0.9993	0.05	0.20
*m*-Toluidine	*y*=1.040*x*+0.065	0.9998	0.02	0.08
2,4-Xylidine	*y*=0.862*x*-0.070	0.9999	0.06	0.24
2,6-Xylidine	*y*=0.856*x*+0.026	0.9997	0.03	0.12
*o*-Anisidine	*y*=0.623*x*+0.018	0.9998	0.06	0.24
*m*-Chloroaniline	*y*=0.354*x*-0.002	0.9997	0.03	0.12
*p*-Chloroaniline	*y*=0.834*x*-0.007	0.9994	0.07	0.28
*m*-Nitroaniline	*y*=0.468*x*-0.135	0.9995	0.05	0.20
2-Naphthalenamine	*y*=1.467*x*-0.125	0.9995	0.03	0.12
*p*-Nitroaniline	*y*=0.462*x*-0.060	0.9998	0.04	0.16
Benzidine	*y*=1.610*x*-0.819	0.9994	0.04	0.16
3,3'-Dichlorobenzidine	*y*=0.830*x*-0.303	0.9994	0.06	0.24
*p*-Chloroaniline-d_2_	*y*=1.330*x*-0.075	0.9997	0.07	0.28

*y*: peak area ratio of the quantitative ion to internal standard; *x*: mass concentration, mg/L.

#### 2.3.2 回收率和精密度

在阴性黄壤土和稻田土实际样品中分别添加含量为1 mg/kg和10 mg/kg的14种目标化合物,进行加标回收试验。稻田土加标前后的总离子流色谱图见图2。重复6次实验,计算加标回收率和相对标准偏差(RSD)。实际样品中苯胺类和联苯胺类化合物的平均加标回收率为62.9%~101.0%,对应的RSD为3.8%~10.3%,相关结果见表6。在相同加标水平下,将本方法与HJ 1210-2021进行比对。根据计算结果,双侧检验统计概率值(*P* value)>显著性水平(*α*)=0.05,表明本方法与HJ 1210-2021无显著性差异(*P*>0.05),精密度和回收率均较好,能够满足土壤中苯胺类和联苯胺类化合物的分析要求。

**图2:**
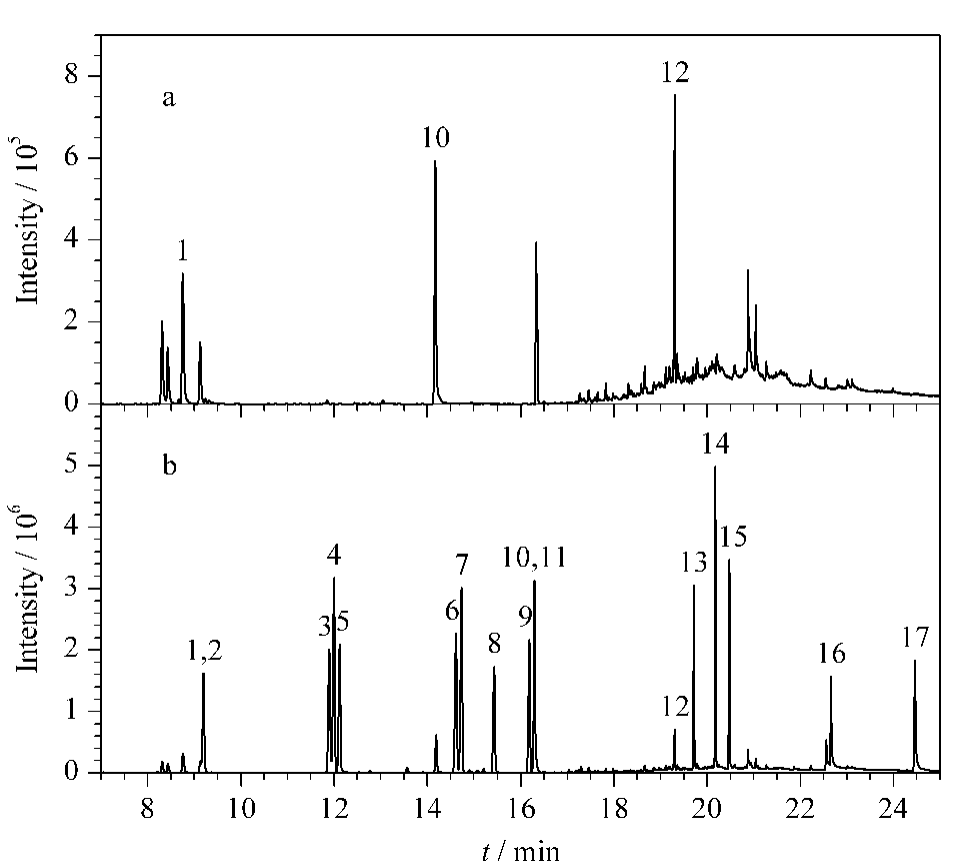
稻田土样品在(a)加标前和(b)加标后的总离子流色谱图

**表6:** 实际土壤样品中14种目标化合物及替代物的回收率和精密度(*n*=6)

Compound	Yellow earth			Paddy soil	
	Recovery/%	RSD/%		Recovery/%	RSD/%
Aniline	71.3	5.7		81.4	9.5
*p*-Toluidine	65.1	3.9		65.9	9.3
*o*-Toluidine	62.9	4.4		83.5	9.3
*m*-Toluidine	68.8	4.3		87.3	9.0
2,4-Xylidine	67.8	3.8		86.2	9.0
2,6-Xylidine	66.9	5.2		71.2	5.9
*o*-Anisidine	74.8	4.2		89.4	9.2
*m*-Chloroaniline	76.9	3.8		88.7	9.4
*p*-Chloroaniline	70.6	6.9		90.9	9.0
*m*-Nitroaniline	76.4	5.9		92.4	9.0
2-Naphthalenamine	71.2	10.3		92.6	8.4
*p*-Nitroaniline	79.2	6.4		94.6	9.6
Benzidine	73.7	8.3		101.0	7.3
3,3'-Dichlorobenzidine	71.1	5.8		88.2	8.6
*p*-Chloroaniline-d_2_	71.5	7.0		88.5	8.5

### 2.4 实际样品分析

利用本文所建立方法对江苏某工业企业疑似苯胺污染地块土壤进行了检测,共检出苯胺及2-甲基苯胺两种化合物,含量分别为1.14 mg/kg和1.27 mg/kg,每个样品重复测定两次,相对偏差(RD)分别为4.4%和5.2%,检出结果与生产调研情况吻合。从现场采样到实验室分析都表明,本方法具有实用、操作易行等特点,能够满足我国土壤中苯胺类和联苯胺类化合物监测分析的要求。

## 3 结论

本文建立了气相色谱-质谱测定土壤中14种苯胺类和联苯胺类化合物的分析方法,优化了样品提取方式、保存及净化方法,评估了方法学指标,并成功应用于实际样品的测定。本方法利用加入抗氧化剂的方式简单、有效地抑制了苯胺类和联苯胺类化合物的氧化,回收率高,稳定性好,操作简便且易于推广,可满足土壤中苯胺类和联苯胺类化合物的测定需求,并为现阶段土壤中该类污染物的调查研究提供了技术支撑。
